# Changes in viscoelastic properties of articular cartilage in early stage of osteoarthritis, as determined by optical coherence tomography-based strain rate tomography

**DOI:** 10.1186/s12891-019-2789-4

**Published:** 2019-09-06

**Authors:** Suguru Nakamura, Mitsuhiko Ikebuchi, Souichi Saeki, Daisuke Furukawa, Kumi Orita, Nobuo Niimi, Yoshito Tsukahara, Hiroaki Nakamura

**Affiliations:** 1 Department of Orthopedic Surgery, Osaka City University Graduate School of Medicine, 1-4-3 Asahi-machi Abeno-ku, Osaka, 545-8585 Japan; 2 Department of Mechatronics Engineering, Faculty of Science and Technology, Meijo University, 1-501 Shiogamaguchi Tempaku-ku, Nagoya, Aichi 468-8502 Japan; 3 Department of Intelligent Mechatronics, Faculty of Systems Science and Technology, Akita Prefectural University, 84-4 Ebinokuchi Tsuchiya, Yurihonjo, Akita, 015-0055 Japan; 4 Nippon Sigmax Co. Ltd., 33rd Floor Shinjuku Oak Tower, 6-8-1, Nishi-Shinjuku, Shinjuku-ku, Tokyo, 163-6033 Japan

**Keywords:** Cartilage, Osteoarthritis, Optical coherence tomography, Viscoelastic property, Strain rate, Attenuation coefficient

## Abstract

**Background:**

Biomechanical changes in articular cartilage are associated with the onset of osteoarthritis. We developed an optical coherence tomography-based strain rate tomography method: stress relaxation optical coherence straingraphy (SR-OCSA). The purpose of this study was to establish an approach for measuring mechanical properties of articular cartilage using SR-OCSA, and to investigate the distribution of viscoelastic properties of articular cartilage in early osteoarthritis.

**Methods:**

Anterior cruciate ligament transection surgery was performed on the left knees of 8–9-month-old New Zealand white rabbits. SR-OCSA was used to visualize and measure the viscoelastic properties of articular cartilage via attenuation coefficient of strain rate (ACSR). Using the same conditions as in the SR-OCSA test, an indentation test was conducted, and relaxation time was measured to evaluate the relationship between ACSR and relaxation time.

**Results:**

SR-OCSA could nondestructively detect and visualize changes in the distribution of viscoelastic properties of articular cartilage in early osteoarthritis. SR-OCSA captured significant increases in ACSR in cartilage at 2 weeks after surgery, when a histologically slight osteoarthritis sign was present. As cartilage degeneration progressed, ACSR increased, whereas relaxation time decreased in a time-dependent manner. Moreover, ACSR negatively correlated with relaxation time. In particular, ACSR was elevated around the tidemark and the elevation tended to move as cartilage degeneration progressed.

**Conclusions:**

SR-OCSA could tomographically and nondestructively detect and visualize changes in the distribution of viscoelastic properties of articular cartilage in early osteoarthritis. The mechanical properties around the tidemark were degraded as cartilage degeneration progressed. Thus, SR-OCSA provides important data needed to understand the biomechanics of early osteoarthritis.

## Background

Osteoarthritis (OA) is the most common form of joint disease in the elderly and causes severe pain and disability. While OA has a multifactorial etiology and ultimately involves the entire joint, its central pathological feature is the progressive loss of articular cartilage [1, 2]. There are currently no effective disease-modifying OA drugs (DMOADs) that can reverse this process when cartilage has been lost [2]. Considering that treatment at the early disease stage is expected to prevent disease progression, early detection and treatment of cartilage degeneration progression are crucial [3]. Historically, OA has been diagnosed by determining characteristic radiographic changes in bone; however, these changes are only visible in the irreversible stage, with substantial cartilage loss and degeneration [4]. Therefore, the establishment of a quantitative method for the early evaluation of cartilage degeneration is important to assess the efficacy of DMOADs and develop appropriate cartilage repair strategies.

During joint loading, the cartilage must withstand high loads while maintaining low friction in order to prevent tissue wear and loss. Thus, mechanical properties and coefficient of friction are important indicators of cartilage function. The cartilage extracellular matrix (ECM) comprises type II collagen, proteoglycan, and water; the structure and composition of ECM are strongly associated with mechanical function of the articular cartilage [5, 6]. During OA onset, compositional changes (e.g., collagen network degradation and proteoglycan loss) and mechanical changes (e.g., decreased stiffness and lubricity) precede morphological changes (e.g., cartilage volume loss) [5]. In the early stages of OA, the collagen network is degraded and proteoglycans decrease, causing mechanical changes [7, 8]. Therefore, identifying mechanical changes in articular cartilage is important for the early diagnosis of OA.

Optical coherence tomography (OCT) is a real-time, noninvasive tomographic imaging method that can be used to capture micrometer-resolution images using near-infrared broadband light [9–11]. OCT detects backscattered rays from inside the object and yields a cross-sectional image with high-resolution of approximately 5 μm, which is comparable to that of low-power histology. It is sensitive to the detection of structural changes caused by trauma and degeneration, and is reportedly useful as a diagnostic instrument for early-stage OA [9–13]. We have developed an OCT-based strain rate tomography method: stress relaxation optical coherence straingraphy (SR-OCSA). Straingraphy is a term combining “strain” and “tomography.” OCSA is a tomographic detection method for the microscale viscoelastic properties of articular cartilage, which mainly comprises a digital image correlation method adapted to OCT images [14]. SR-OCSA can visualize the distribution of viscoelastic properties tomographically and nondestructively under ex vivo experimental conditions; thus, it may reveal biomechanics in articular cartilage during early stage OA by detecting characteristic viscoelastic properties.

This study aimed to visualize and evaluate the distribution of viscoelastic properties in articular cartilage of early OA in a rabbit model of OA by using SR-OCSA under ex vivo experimental conditions. Furthermore, an indentation test using a universal tabletop mechanical tester under the same SR-OCSA conditions was conducted to verify the validity of the mechanical properties determined by SR-OCSA.

## Methods

### Animal model

Female New Zealand white rabbits aged 8–9 months and weighing 3.6–4.0 kg with closed epiphyses (SLC, Inc. Japan) were used for this study. Closed epiphyses were confirmed via radiography. Experimental OA was induced via anterior cruciate ligament transection (ACLT) in the left knee joint. All rabbits were anesthetized with subcutaneous injection of ketamine (40 mg/kg) and xylazine (5 mg/kg). The medial parapatellar incision was made, and the patella was dislocated laterally. The ACL was visualized and transected with a No. 11 blade. An anterior drawing test was performed to confirm that the ACL was transected completely. The joint was irrigated with sterile saline, and the capsule and skin were closed. Postoperatively, the animals with free activity allowed were housed singly in cages in an animal room with controlled temperature and humidity and periodic light cycles. The animals were sacrificed at 2, 4, and 8 weeks after surgery (n = 5/time point) via intravenous injection of sodium pentobarbital (3 ml). Three animals who did not undergo ACLT were sacrificed for the control group (n = 6). After euthanasia, distal femurs were amputated at the supracondylar site using an electric saw. Distal femurs were then prepared for mechanical tests and histological analysis. All animal experiments were strictly performed in accordance with the regulations of the Institutional Animal Care and Use Committee, Osaka City University Medical School (Approval number: 15012).

### SR-OCSA mechanical test

**Fig. 1:**
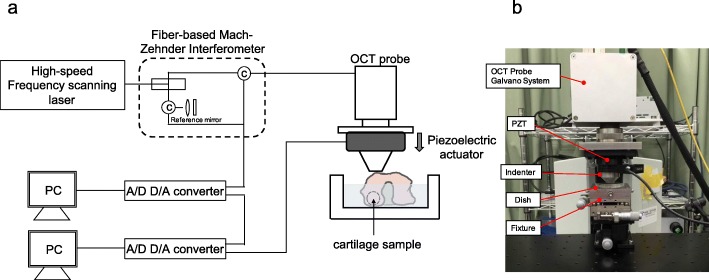
Stress relaxation optical coherence straingraphy (SR-OCSA) system. **a** Illustration of the experimental apparatus. **b** Photograph of the system

**Fig. 2:**
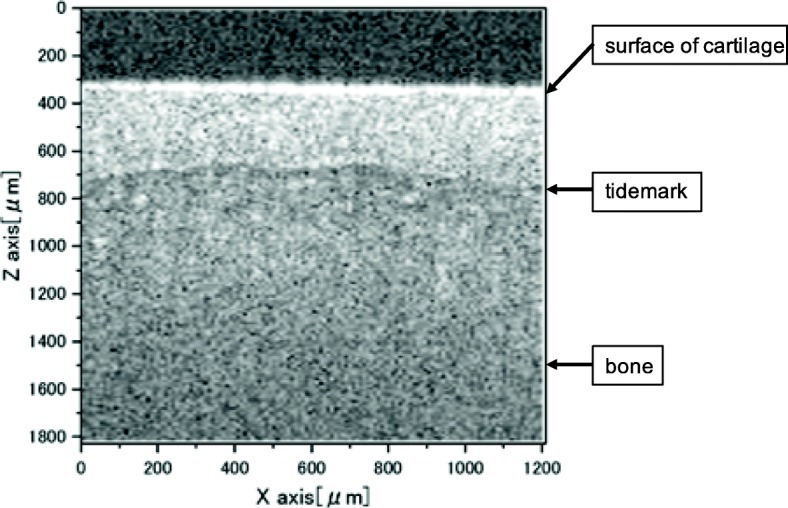
Optical coherence tomography (OCT) image of articular cartilage. The highest signal line, around 300 μm depth, is surface of cartilage. The second highest signal line, around 700 μm depth, is the boundary between non-calcified cartilage and calcified cartilage (tidemark)

The SR-OCSA system is shown in Fig. 1a and b. The SR-OCSA system was constructed with the Swept Source OCT (IVS-2000; Santec Inc., Aichi, Japan) system by taking the video rate of tissue tomogram and an indenter controlled by a piezoelectric (PZT) actuator (nPFocus100; nPoint Inc., Middleton, MI, USA). Mechanical measurement locations were defined as the top point of the femoral condyle. The distal femurs were glued onto a dish using cyanoacrylate adhesive gel and immersed in phosphate-buffered saline. The dish was fixed to the stage using a handmade fixture. The cartilage image was acquired by using OCT, and the cartilage thickness was measured from the acquired OCT image (Fig. 2). The actuator was driven from the state where the indenter was in close contact with the cartilage at the same location as the OCT image experiment was conducted. In the stress relaxation test, the amount of cartilage compression was set to 10% of the thickness of articular cartilage. All samples were tested using a stress-relaxation protocol (10% pre-strain, 10% strain, 2.5% strain per second speed, and 60 s relaxation time). The deformed cartilage was visualized using the OCT probe installed on the upper side of the revolution arm while deforming with the indenter. The system had a depth (*z*) and width (*x*) resolution of 7.1 μm and 7.5 μm, respectively. The OCT image size acquired was set as a 2-dimensional *xz* tomographic image of 1 mm × 1.51 mm (128 × 192 pixel), and images were continuously acquired at 79.05 frames per second (a total of 4743 frames). By obtaining the image acquisition signal of the OCT and the drive signal of the PZT actuator, the compression relaxation behavior and OCT tomographic image can be synchronized. In this approach, OCT images are captured continuously during the stress relaxation test using a PZT actuated indenter, and SR-OCSA can provide the tomography of the deformation vector and strain rate.

### SR-OCSA algorithm

**Fig. 3:**
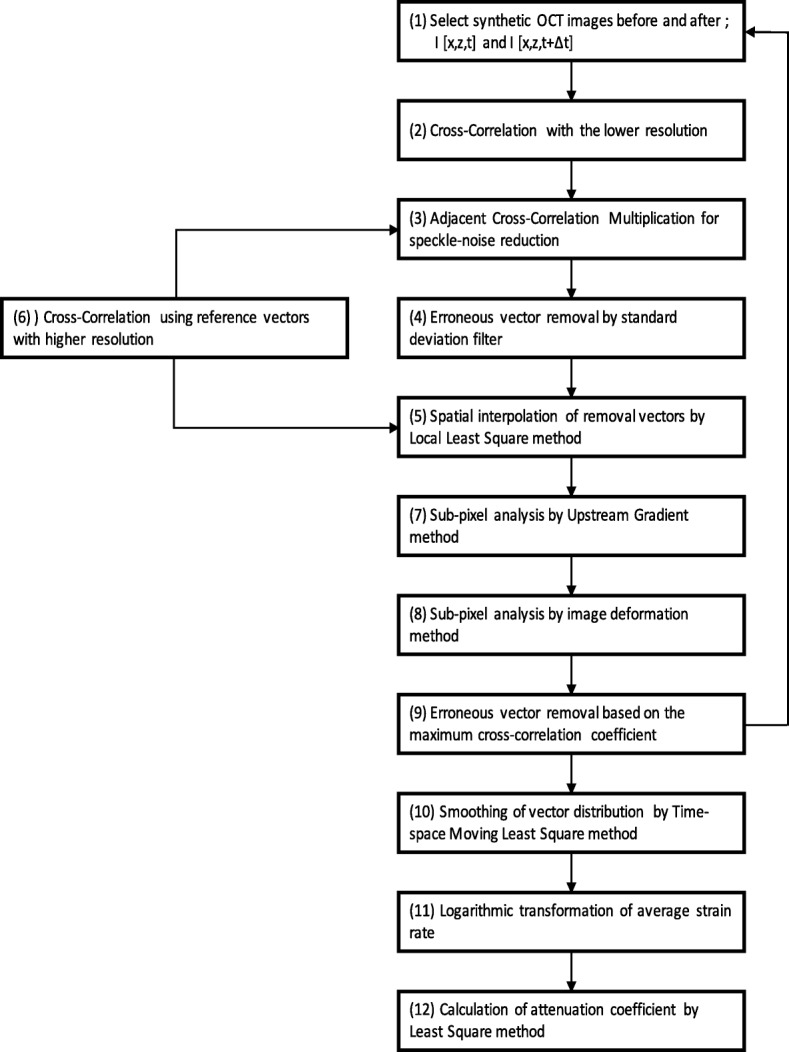
Flowchart of optical coherence straingraphy (OCSA) algorithm

**Fig. 4:**
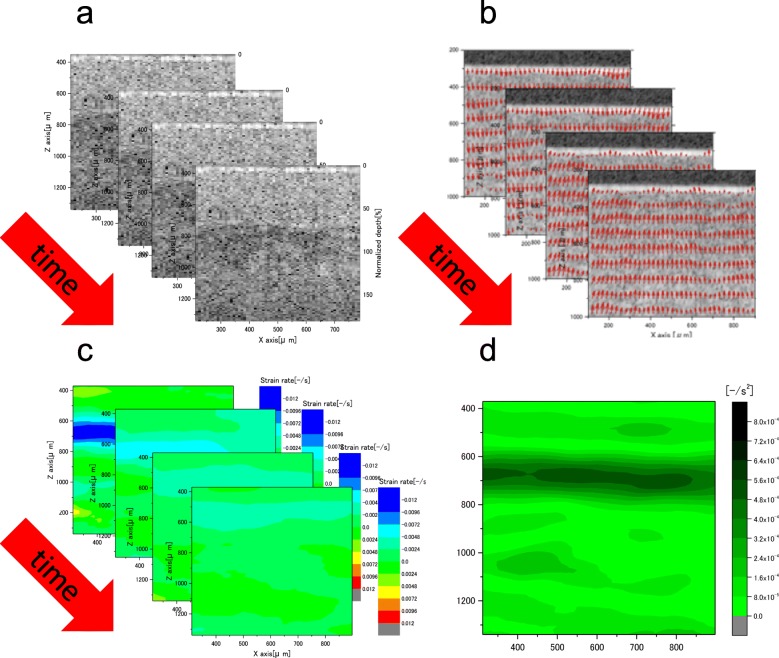
**a** shows consecutive optical coherence tomography (OCT) images during stress relaxation (corresponds to OCSA algorithm No. 1). **b** shows tomographic images of the speed vector generated by tissue deformation. This data is obtained through an algorithm OCSA algorithm No. 2–9. **c** shows tomographic distribution of the strain rate. This data is obtained through an algorithm OCSA algorithm No. 10. **d** shows Tomographic distribution of attenuation coefficient of strain rate (ACSR) during stress relaxation. This data is obtained through an algorithm OCSA algorithm No. 11–12

OCSA is a new method to detect the mechanical properties at the microscale using OCT, and it is mainly constructed using a digital image correlation method to tomographically detect the displacement vector distribution inside tissues [14]. Specifically, the OCSA is applied to the OCT tomographic images obtained under mechanical load to the tissue before and after deformation. OCSA directly detects the displacement vector distribution of the tissue and was continuously performed in this study on the acquired continuous OCT images to tomographically detect the strain rate distribution inside the tissue at the microscale. The flowchart of the OCSA algorithm is shown in Fig. 3. The tissue is deformed by the mechanical load from the actuator, and the OCT tomographic images before and after deformation are acquired (Fig. 4a). The OCSA algorithm uses the speckle cross-correlation analysis recursively to obtain synthetic OCT images with a quarter reduction of the subset window size. However, OCT images have speckle noise and contain less valid information than other images commonly used in digital image correlation (DIC) analysis. Furthermore, an OCT image typically has the lower number of pixels than a normal DIC image; such low quality and quantity cause erroneous vectors in cross-correlation analysis. As shown in Fig. 3, the cross-correlation method and adjacent cross-correlation multiplication technique are introduced recursively to correct vectors and enhance the vector resolution by suppressing the erroneous vectors and error propagation. Furthermore, the amount of high-precision subpixel deformation is calculated using the sub-pixel analysis method (the upstream gradient method and image deformation method). OCSA can provide data on the distribution of highly accurate speed vectors, taking account of tissue deformation, as an instantaneous tomographic image (Fig. 4b). The time sequential data of the vector field are smoothed by the time-space moving least square method as the approximated speed vectors. The tomographic distribution of the strain rate tensor is calculated from the differential coefficient of the approximate expression (Fig. 4c).

The viscoelastic body shows stress relaxation behavior when the whole body is held and the stress gradually decreases. The micro deformation inside the viscoelastic body is observed with stress relaxation. Therefore, we adopted the SR-OCSA (i.e., continuous OCSA) during the stress relaxation test to detect sequential tomographic changes in the strain rate. Linear approximation of *α (x, z, t) t + β (x, z, t)* was applied to the detected compressive strain rate in the depth direction (z) to visually evaluate the viscoelastic properties of the tissue as attenuation coefficient of strain rate (ACSR). ACSR is an index of the viscoelastic properties during stress relaxation (Fig. 4d). The viscoelastic properties in the object were determined from the ACSR averaged in the cartilage region determined from the OCT image.

### Indentation test

After the SR-OCSA experiment, the validity of the ACSR obtained from SR-OCSA was verified via an indentation test using a universal tabletop mechanical tester (EZ-LX; Shimadzu, Kyoto, Japan) under the same conditions as those in the SR-OCSA mechanical test (10% pre-strain, 10% strain, 2.5% strain/sec speed, and 60 s relaxation time). The compressive forces were measured instantaneously via the load cell, and the force relaxation curves were acquired during the indentation test and were approximated using an exponential decay function. Relaxation time (*τ*) was defined as the time at which the initial stress becomes 1/*e* in stress relaxation and represents the ratio of the viscosity and elasticity of the viscoelastic body, i.e., the balance of elasticity and viscosity.

### Histological cartilage assessment

The samples were fixed with 4% paraformaldehyde and decalcified by soaking in Morse’s solution for 2 weeks. The samples were then dehydrated and embedded in paraffin and were cut in 4-μm microsections in the sagittal plane of the medial femoral condyle. The sections were deparaffinized using xylene and ethanol and stained with hematoxylin/eosin and safranin O-fast green to evaluate OA changes. Histological changes of the articular cartilage in the medial femoral condyle were assessed using the Osteoarthritis Research Society International (OARSI) histochemical/histological scoring system [15]. This grading system assigns scores ranging from 0 to 24 based on the safranin O staining: staining (0–6), structure (0–11), chondrocyte density (0–4), and cluster formation (0–3).

### Statistical analyses

One-way analysis of variance test with Tukey’s post hoc analysis to compare the averages of continuous variables among the four groups was used. The correlations between *τ* and ACSR, OARSI score and *τ*, and OARSI score and ACSR were tested using the Spearman correlation analysis test. Statistical analyses were performed with GraphPad Prism v.7.0. (GraphPad Software, La Jolla, Ca). P < 0.05 was considered significant. Post hoc power analysis verified that the experimental protocol could distinguish between groups with a statistical power of > 0.80. The post hoc power analysis was performed using G*power software (Faul, Erdfelder, Lang & Buchner, 2007).

## Results

### Histological assessment of the cartilage

**Fig. 5:**
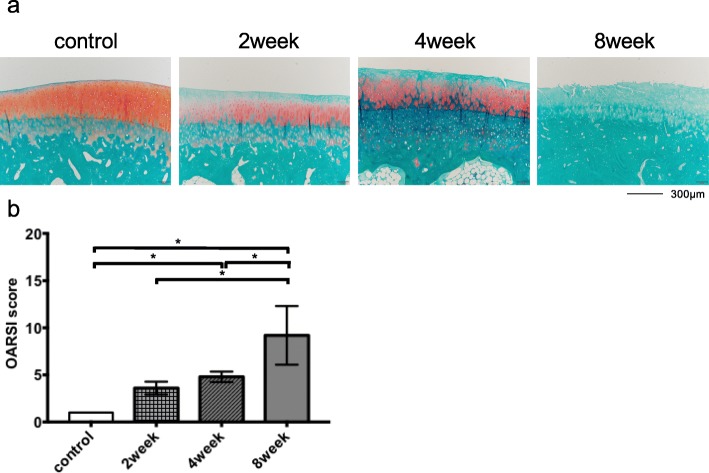
**a** Representative Safranin O-fast green stained histological section of each group. **b** Osteoarthritis Research Society International (OARSI) histological score in the control (*n* = 6) and the anterior cruciate ligament transection (ACLT) rabbit osteoarthritis (OA) model (each model, *n* = 5). Values are presented as the means ± standard deviations (error bars). *P < 0.01, compared between groups

At 2 weeks after ACLT, there were slight OA signs, intact surface, and reduction in safranin O staining only in the surface layer (Fig. 5a). At 4 weeks after ACLT, there were apparent OA signs, including superficial diffuse loss of safranin O and fibrillation. At 8 weeks after ACLT, there was diffuse loss of safranin O in the whole layer and fibrillation extending to the middle zone. The OARSI histological scores were 3.4 ± 0.5 at 2 weeks after ACLT (n = 5, mean ± standard deviation), 4.8 ± 0.4 at 4 weeks after ACLT (n = 5), and 9.2 ± 2.8 at 8 weeks after ACLT (n = 5; Fig. 5b). There were no histological OA signs in the control group. The OARSI histological score in the control group was 1.0 ± 0 (n = 6). The OARSI histological scores at 2 weeks after ACLT were higher than in the control group, but the difference was not significant (control vs 2 weeks: p = 0.06; Fig. 5b). The OARSI score significantly increased in a time-dependent manner, beginning at 4 weeks after ACLT (control vs 4 weeks: p < 0.01, control vs 8 weeks: p < 0.01, 2 weeks vs 4 weeks: p = 0.62, 2 weeks vs 8 weeks: p < 0.01, and 4 weeks vs 8 weeks: p < 0.01; Fig. 5b).

### Mechanical test

**Fig. 6:**
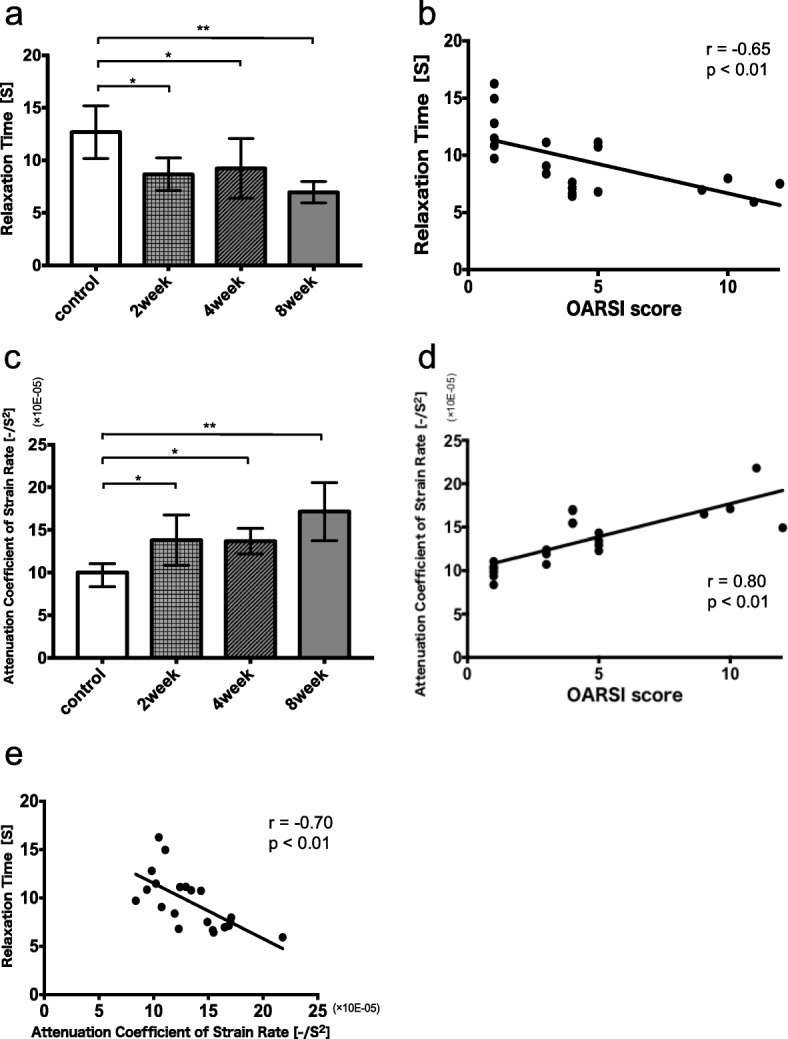
**a** Relaxation time (*τ*) of each group. **b** Correlation between Osteoarthritis Research Society International (OARSI) score and *τ*. **c** Averaged attenuation coefficient of strain rate (ACSR) of each group. **d** Correlation between OARSI score and ACSR. **e** Correlation between *τ* and ACSR. Values are presented as the means ± standard deviations (error bars). *P < 0.05, **P < 0.01 compared between groups

The mechanical properties of the cartilage measured via the universal tabletop mechanical tester are summarized in Fig. 6a. The relaxation time (*τ*) in the control group showed the highest value (12.7 ± 2.3 s, n = 6) (mean ± S.D.). At 2 weeks after ACLT, *τ* significantly decreased, although the histological change was not prominent (2 weeks: 8.7 ± 1.4 s, n = 5), and *τ* in the ACLT groups tended to decrease in a time-dependent manner as histological changes became more prominent (4 weeks: 9.2 ± 2.0 s, n = 5; 8 weeks: 7.0 ± 0.7 s, n = 5) (control vs 2 weeks: p = 0.02, control vs 4 weeks: p = 0.04, control vs 8 weeks: p < 0.01). Furthermore, *τ* was negatively correlated with the OARSI score (r = − 0.65, p < 0.01; Fig. 6b).

### SR-OCSA mechanical test

Similar to the relaxation time, at 2 weeks after ACLT, the averaged ACSR in the cartilage significantly increased much earlier than when the histological change became prominent (control vs 2 weeks: p = 0.03; Fig. 6c). ACSR in the ACLT groups tended to increase in a time-dependent manner as histological changes became more prominent (control vs 4 weeks: p = 0.04; control vs 8 weeks: p < 0.01). Furthermore, ACSR had a strong positive correlation with the OARSI score (r = 0.80, p < 0.01; Fig. 6d) and a strong negative correlation with *τ* (r = − 0.70, p < 0.01; Fig. 6e).

**Fig. 7:**
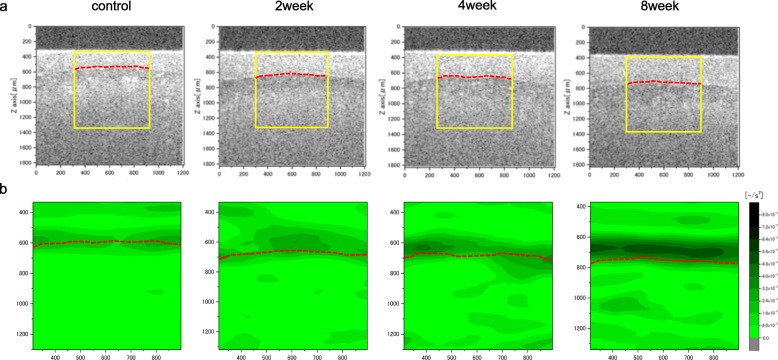
Representative optical coherence tomography (OCT) image of each group (**a**) and corresponding tomographic distribution of attenuation coefficient of strain rate (ACSR) in the same sample (**b**). The yellow box is the measurement range of stress relaxation optical coherence straingraphy (SR-OCSA) and the red dotted line is the tidemark. Each group showed elevation of the ACSR around tidemark and the degree of elevation in the ACSR increased in a time-dependent manner

A representative OCT image and the distribution of ACSR in each group are shown in Fig. 7a and Fig. 7b. The elevation of ACSR is expressed via becoming darker in the color tone. Each group showed mainly elevated ACSR around the tidemark, rather than in the superficial layer. Compared with the control group, the group at the 2 weeks after ACLT showed an expansion of the range where ACSR in the deep layer increase. The group at 4 and 8 weeks after ACLT showed further increase in ACSR in the deep layer. The degree of elevation in ACSR showed a tendency to be larger in a time-dependent manner.

## Discussion

We developed an OCT-based straingraphy, SR-OCSA, as a tomographic method for visualizing viscoelastic properties of articular cartilage. Our study showed that the SR-OCSA could nondestructively detect changes of viscoelastic properties in degenerative cartilage via tomographic distribution, which was associated with degenerative cartilage changes in the rabbit OA model. These findings support the use of SR-OCSA as a new measurement tool to evaluate the biomechanics of articular cartilage.

As shown in Fig. 5 and Fig. 6a and b, cartilage with an increasing OARSI score had reduced viscoelastic properties for withstanding compressive loads [7]. Structural and biochemical changes in articular cartilage occur throughout the pathogenesis of OA [16, 17]. Early OA changes include an increase in water content due to proteoglycan loss and disorganization of the type II collagen network [18–20]; these changes cause reductions in the mechanical properties of cartilage in the early stages of OA [7, 18, 19, 21, 22]. Type II collagen mainly contributes to the tensile modulus and the dynamic properties of the cartilage, while proteoglycans are primarily responsible for the equilibrium response in compression [18, 23, 24]. Indentation-type atomic force microscopy on the surface of the cartilage has shown that the viscoelastic properties are decreased in OA cartilage, compared with healthy cartilage [22].

Our mechanical experiment showed that *τ* in the whole articular cartilage of the rabbit OA model tended to change in a time-dependent manner after surgery (Fig. 6a), and was significantly correlated with the OARSI score (Fig. 6b). Consistent with the findings of a previous study [22], our study showed the further reduction of *τ* in OA cartilages as compared with healthy ones. Our result indicates that OA cartilages have lower viscosity than healthy ones. Additionally, *τ* significantly correlated with the OARSI score, which is consistent with the findings of previous studies examining the relationship between mechanical properties in osteoarthritic cartilage and the histological score.

As shown in Fig. 6c and d, ACSR from SR-OCSA in the articular cartilage of the rabbit OA model tended to change in a time-dependent manner after surgery, and was strongly correlated with the OARSI score. As shown in Fig. 6e, ACSR from SR-OCSA was negatively correlated with *τ* from the universal mechanical tester under the same conditions. Thus, SR-OCSA may provide a mechanical index of viscoelastic properties; moreover, SR-OCSA could detect changes in viscoelastic properties associated with degenerative cartilage changes in the rabbit OA model.

A previous study reported a significant difference in viscoelastic properties between cartilage without signs of degeneration and cartilage with early OA signs [7, 8]. SR-OCSA could capture the significant increase of ACSR at 2 weeks after ACLT with an intact surface and a histologically slight OA sign. This result also indicates that viscoelastic properties (i.e., ACSR) can serve as a sensitive indicator of OA sign in cartilage. Therefore, from the mechanical perspective, SR-OCSA has sufficient sensitivity to detect slight degeneration of cartilage during early OA onset.

SR-OCSA could nondestructively capture the tomographic distribution of viscoelastic properties in micrometer resolution and show the accompanying degenerative changes in OA cartilage. As shown by Fig. 7b, it was possible to evaluate the tomographic map of viscoelastic degeneration in the tissue by matching with the OCT image (Fig. 7a). Specifically, as cartilage degeneration progressed, we could visually confirm that ACSR increased and its range expanded. In this way, the SR-OCSA was able to visually distinguish the difference of viscoelastic properties. A previous study reported that OCT is a useful diagnostic tool for early OA by capturing tissue structural changes in the cartilage [9]. However, the image using the OCT is only the morphological tissue image. Thus, OCT can detect OA only after a superficial cartilage has been damaged. SR-OCSA can capture not only the morphological tissue image, but also the spatial and temporal change of tissue deformation during the stress relaxation, i.e. the distribution of strain rate, whereby a tomographic image of mechanical properties can be provided as a viscoelastic degeneration map predisposed to OA even before a superficial cartilage has been damaged.

To our knowledge, SR-OCSA is a new measurement tool that enables tomographic visualization of viscoelastic properties in overall cartilage layer at the micrometer scale. Figure 7b additionally shows an elevation of the ACSR in cartilage, particularly in the deep zone around the tidemark. Moreover, the degree of elevation in the ACSR tended to increase in a time-dependent manner. The degradation of mechanical properties of cartilage in early OA has been reported via an indentation-type atomic force microscopy applied on the cartilage surface [8, 22]. However, to the best of our knowledge, no study has been conducted on nondestructive analysis of the viscoelastic properties of the entire articular cartilage, including the deep layer.

Our results indicate that viscoelastic properties are degraded in the deep layer around the tidemark as cartilage degeneration progresses. Previous reports have shown that the equilibrium confined compression modulus of articular cartilage increases significantly with distance from the articular surface [25]; moreover, significant changes of collagen content and collagen angle in the deep layer and volume loss of subchondral bone and trabecular bone were observed at 4 weeks after ACLT in the rabbit OA model [26, 27]. In addition, modulus in the zone of calcified cartilage and subchondral bone was altered in early OA [28]. Because of the large mechanical loading and compositional change in the deep layer, it is highly possible that the mechanical properties are substantially degraded in the deep layer around the tidemark as cartilage degeneration progresses. However, we are the first to report such findings; thus, they should be verified in further studies.

Conventional methods of measuring cartilage mechanical properties in the deep layer required removing the cartilage sample from its native environment, which may damage the continuum structure of the tissue and alter the mechanical properties. On the other hand, SR-OCSA can nondestructively measure the mechanical properties in the deep layer without damaging the cartilage, making it possible to evaluate in a situation closer to the native environment than the conventional methods. Because SR-OCSA can nondestructively measure the tomographic distribution of viscoelastic properties, which was impossible via the conventional mechanical test, it can be applied to the articular cartilage, as well as to various biological samples for evaluating the pathophysiology and biomechanics of other diseases.

This study has some limitations. First, we used an experimental surgical instability model of OA in rabbits induced by the ACL transection. The ACLT OA model in rabbits is a reproducible and effective model of OA. However, this OA model may be different from natural history OA. Second, SR-OCSA is based on OCT, so the measurement range is limited due to the limited light permeability. Because rabbit cartilage can be evaluated as thin as 400–500 μ, SR-OCSA could evaluate cartilage deep layer and subchondral bone. But in the case of human thick articular cartilage, it may not be possible to analyze deep layers or subchondral bone due to this limitation.

## Conclusions

In summary, SR-OCSA is a useful mechanical instrument that can allow for a nondestructive tomographic visualization of the viscoelastic properties in the cartilage. Particularly, it can detect the spatial changes in the ACSR due to viscoelastic degeneration in osteoarthritic cartilage of the rabbit OA model. Using SR-OCSA, we found that the viscoelastic properties of the cartilage are degraded in the deep layer around the tidemark as cartilage degeneration progresses. SR-OCSA provides important information regarding the biomechanics of early OA, which can be used for the early detection of OA and development of appropriate prevention and treatment strategies.

## Data Availability

The datasets used and/or analyzed during this study are available from the corresponding author on reasonable request.

## Abbreviations

ACLT: Anterior cruciate ligament transection
ACSR: Attenuation coefficient of strain rate
OA: Osteoarthritis
OARSI: Osteoarthritis Research Society International
OCT: Optical coherence tomography
SR-OCSA: Stress relaxation optical coherence straingraphy
